# TFBSshape: a motif database for DNA shape features of transcription factor binding sites

**DOI:** 10.1093/nar/gkt1087

**Published:** 2013-11-07

**Authors:** Lin Yang, Tianyin Zhou, Iris Dror, Anthony Mathelier, Wyeth W. Wasserman, Raluca Gordân, Remo Rohs

**Affiliations:** ^1^Molecular and Computational Biology Program, University of Southern California, Los Angeles, CA 90089, USA, ^2^Department of Biology, Technion – Israel Institute of Technology, Technion City, Haifa 32000, Israel, ^3^Centre for Molecular Medicine and Therapeutics, University of British Columbia, Vancouver, BC, Canada and ^4^Institute for Genome Sciences & Policy, Duke University, Durham, NC 27708, USA

## Abstract

Transcription factor binding sites (TFBSs) are most commonly characterized by the nucleotide preferences at each position of the DNA target. Whereas these sequence motifs are quite accurate descriptions of DNA binding specificities of transcription factors (TFs), proteins recognize DNA as a three-dimensional object. DNA structural features refine the description of TF binding specificities and provide mechanistic insights into protein–DNA recognition. Existing motif databases contain extensive nucleotide sequences identified in binding experiments based on their selection by a TF. To utilize DNA shape information when analysing the DNA binding specificities of TFs, we developed a new tool, the TFBSshape database (available at http://rohslab.cmb.usc.edu/TFBSshape/), for calculating DNA structural features from nucleotide sequences provided by motif databases. The TFBSshape database can be used to generate heat maps and quantitative data for DNA structural features (i.e., minor groove width, roll, propeller twist and helix twist) for 739 TF datasets from 23 different species derived from the motif databases JASPAR and UniPROBE. As demonstrated for the basic helix-loop-helix and homeodomain TF families, our TFBSshape database can be used to compare, qualitatively and quantitatively, the DNA binding specificities of closely related TFs and, thus, uncover differential DNA binding specificities that are not apparent from nucleotide sequence alone.

## INTRODUCTION

The DNA binding specificities of transcription factors (TFs) can be described as consensus sequences or position frequency matrices (PFMs) representing the probability of occurrence of each nucleotide at each position of a DNA binding site. These probability matrices are usually transformed into position weight matrices (PWMs) (1,2) and can be visualized as motif logos (3). PWMs traditionally assume independence between individual nucleotide positions within the binding site. Recent approaches have expanded the basic concept of PWMs by adding dinucleotide parameters, based on observations that individual nucleotide positions within a motif are not independent from each other (4–6). Interdependencies between nucleotide positions within a motif give rise to the three-dimensional structure of DNA and, thus, the shape of TF binding sites (TFBSs).

The important role of DNA shape as a determinant of protein–DNA binding specificity has been previously discussed (7–10), and we have demonstrated mechanisms of DNA shape readout for numerous TFs (11–17) and other DNA binding proteins (18–20). The DNA structure implicitly contains all interdependencies between nucleotide positions of a TFBS and does not require explicit knowledge of individual interdependencies. Although DNA shape is a function of sequence, the sequence–structure relationship is highly complex and degenerate. DNA shape can explain why sequences that flank TFBSs contribute to binding specificity (17). Spacers between binding sites of different DNA binding proteins (21) or half sites of multimeric TFs can play a similarly important role (22,23).

Structural data have only been obtained for a small number of relatively short DNA sequences that have been studied experimentally or by computationally expensive molecular simulations. This limited availability has been a major bottleneck for using DNA shape information in genome analysis. To overcome this limitation, we recently developed a fast and efficient method for the high-throughput prediction of DNA shape, and validated the method with massive experimental and computational data (24).

Using this approach, in the present study, we describe the development of our TFBSshape database, which provides DNA structural features for nucleotide sequences preferred by different TFs. We analysed 739 datasets derived from open-access motif databases that describe the DNA binding specificities of TFs from 23 different species. We used the sequence information provided by JASPAR (25) and UniPROBE (26) to calculate DNA shape features of TFBSs. These features include minor groove width (MGW), Roll, propeller twist (ProT) and helix twist (HelT). Our TFBSshape database qualitatively illustrates the TFBS shape profiles in heat maps for TF core binding sites. Flanking sequences are included whenever such information is available. Download options provide quantitative data for further analysis. TFBSshape includes a tool to compare, both qualitatively and quantitatively, any two selected TFBS shape profiles. A user can also upload a sequence dataset and compare its DNA shape features with any chosen TFBS shape profile in the database.

We applied the TFBSshape approach to different biological applications. We analysed the differential DNA shape preferences of the human basic helix-loop-helix (bHLH) TFs Mad2 (‘Mad’), Max and c-Myc (‘Myc’) using genome-context protein binding microarray (gcPBM) data (27). To demonstrate the added value of describing TFBSs using structural features, we used L2-regularized multiple linear regression (MLR) to predict the DNA binding specificities of these bHLH factors based on nucleotide sequence alone compared to a model that combines DNA sequence and shape. We showed that shape-augmented MLR models improved the accuracy in DNA binding specificity predictions by >20%. We also described the DNA shape preferences of Hox proteins in mouse using DNA binding sequences derived from universal protein binding microarray (PBM) experiments (28). The results of this analysis showed that distinct DNA shape features of TFBSs for anterior versus posterior Hox TFs, as previously reported for *Drosophila* (12), can be observed across species.

## DATABASE

### Database architecture and methodology

TFBSshape derives TFBS sequence information from the motif databases JASPAR (25) and UniPROBE (26) and generates DNA shape data for TFBSs based on the high-throughput prediction of DNA structural features, including the parameters MGW, Roll, ProT and HelT (24). The approach uses a sliding pentamer window and query tables of structural features derived from all-atom Monte Carlo simulations for all 512 unique pentamers. We previously validated this method using massive experimental data from X-ray crystallography, NMR spectroscopy and hydroxyl radical cleavage experiments, as well as statistical analysis and cross-validation (24).

The backend of TFBSshape consists of a MySQL database, PHP scripts hosted on an Apache server, and other scripts invoked by the PHP scripts to perform TFBS assembly and DNA shape prediction upon user request (Supplementary Figure S1). The frontend of TFBSshape includes HTML web pages with components of CSS and JavaScripts that provide a user-friendly interface for retrieving data from the database. In addition, it provides an interface for comparison of two TFBS shape profiles from the database and an interface for generating DNA shape data for user-uploaded TFBS sequences, which can also be compared to a TFBS shape profile in the database. Among the TFBS data derived from JASPAR and UniPROBE, 371 TFs are from JASPAR (29), including 149 TFs from the latest JASPAR2014 release (25) and 368 TFs are from UniPROBE (26). TFs in JASPAR or UniPROBE without TFBS sequence information are not included in TFBSshape. Due to their different storage formats, sequence data from JASPAR and UniPROBE need to undergo different pre-processing steps prior to TFBS assembly and DNA shape prediction.

### Interface with JASPAR

JASPAR curates TFBS sequence data derived from the literature in its sub-database JASPAR CORE (29). For TFs with available motif information, the TFBS sequences are provided in FASTA format, with the core binding site highlighted in upper-case letters and the flanking sequences in lower-case letters. Using this sequence data, TFBSshape derives DNA shape features for all nucleotide positions within the core binding site. This prediction is possible because TFBS sequences from JASPAR always contain the core binding sites, whereas their flanking sequences can be missing. Due to the methodology used to predict DNA shape features at the centre of a sliding pentamer (24), 2-bp flanks are needed to calculate the structural features for the entire core binding motif.

Because the flanking sequence information can be missing, TFBSshape first calculates the portion of TFBS sequences that contain ≥2-bp flanks on each side of the core binding site. If this portion is >50% of all sequences available for a TF, sequences that do not contain 2-bp flanks are removed, and the TFBSs are assembled in the form of nnNNN … NNNnn using the remaining sequences. Here, the stretch of NNN … NNN represents the core binding site, and nn represents the 2-bp flanks required for predicting DNA shape features for the two NN positions at either end of the core binding site. If the portion of TFBSs with flanking sequence information is ≤50%, then no sequences are removed, and TFBSs are assembled in the form of NNN … NNN. In this case, DNA structural features cannot be predicted for the first and last two NN positions of the core binding site.

**Figure 1.**
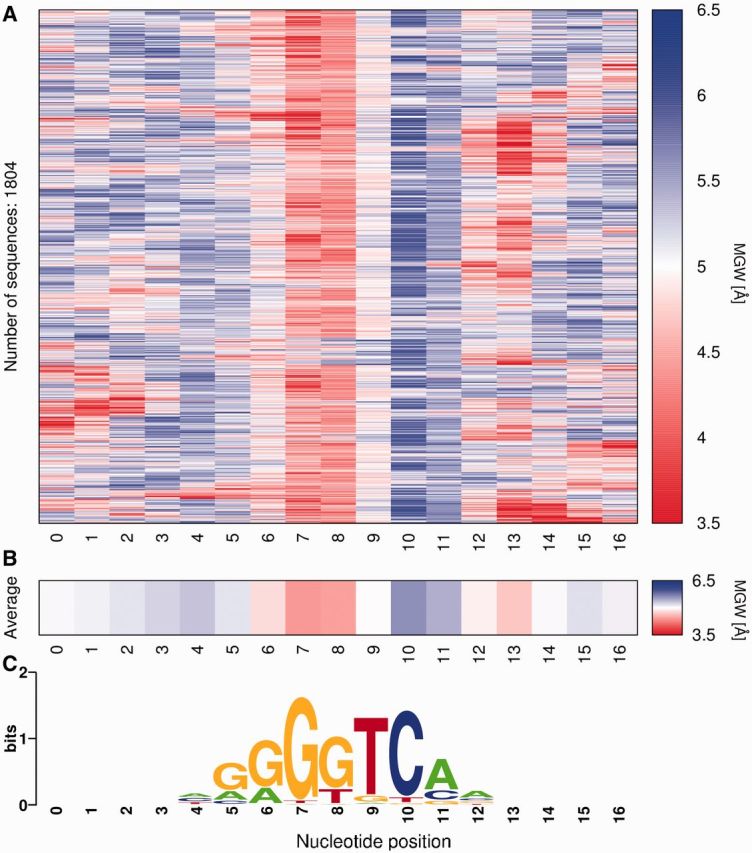
Example TFBSshape analysis of DNA shape preferences for an Hnf4a TF dataset from UniPROBE. (**A**) Heat map showing predicted MGW profiles for individual sequences, clustered based on EDs of MGW profiles, and (**B**) average heat map for all sequences. The colour code for both heat maps uses red for narrow MGW, blue for wide MGW and white for intermediate values. (**C**) PWM calculated using all analysed TFBS sequences, aligned with DNA shape heat maps as nucleotide sequence reference.

In either case, DNA shape features are predicted for the core binding sites and visualized as heat maps. A white space at the left and right margins of the heat maps indicates that structural features for the end positions of the core binding site are not available due to missing flanks. This situation is more common for TFBS data from the previous JASPAR release (29). It does not happen for data from the JASPAR2014 update (25). For each structural feature, a heat map for individual sequences and an average heat map are provided. Each row in the heat map for individual sequences represents the structural feature values of a corresponding sequence (Figure 1A). If the number of rows is ≤3000, these rows are clustered using the agglomerative hierarchical clustering algorithm, with distances defined as the Euclidean distance (ED) between the values of the individual rows. The average heat map provides average structural features at each nucleotide position of the TFBS (Figure 1B). Although these heat maps can be seen as a qualitative analysis, links are provided for the user to download the actual DNA shape data for further quantitative analysis. The PWM generated based on the analysed set of sequences is visualized in the TFBSshape database as a motif logo (Figure 1C). In this format, the numbering of the nucleotide positions corresponds to the numbering used in the structural feature heat maps.

### Interface with UniPROBE

UniPROBE hosts TF binding data generated from universal PBM experiments (26). In these experiments, each array is designed to contain probes that cover all possible 10-mer variants (30). A TF of interest can access each probe to initiate a binding event, and the binding intensities for different probes are compared based on their fluorescence signal intensities. These data are used to derive PFMs that represent the *in vitro* DNA binding specificities of the TF. TFBSshape retrieves the probe set and PFM from UniPROBE and uses them as input data of the Find Individual Motif Occurrences (FIMO) algorithm (31) from the MEME Suite of motif-based sequence analysis tools (32). FIMO searches in the input sequences for occurrences of a motif specified by a PFM. Each motif occurrence found by FIMO is associated with a *P*-value*,* indicating the statistical significance level of the detected occurrence. TFBSshape determines motif occurrences with *P* ≤ 10^−^^3^ as core binding sites. This significance level is an empirical threshold based on the fact that PFMs generated from such TFBSs are highly consistent with the original PFMs provided by UniPROBE (26).

The assumption that TFBSs are enriched among probes with higher PBM signal intensities is generally true for TFBSs found in the above manner. A barcode visualizes the enrichment of the TFBSs among the ranked probes, with vertical bars representing probes with PBM signal intensities in descending order from left to right, and a white bar indicating no occurrence of a TFBS, a yellow bar indicating one TFBS and a brown bar indicating multiple TFBSs. TFBS-containing probes are subjected to the same TFBS assembly and other procedures as described for JASPAR. TFBSs derived from UniPROBE data usually have ≥2-bp flanks because most TFBSs are not located at the end of the probe. The TFBS sequence and shape data can be downloaded for further quantitative analysis.

### User interface for analysis of DNA shape profile of one TF dataset

TFBSshape provides tab pages that dynamically display specific content and form an interface for retrieving data. The ‘Selection’ tab enables the user to specify the search criteria for either JASPAR or UniPROBE data or to upload custom-aligned sequences. After the user initiates the selection, the ‘Refine’ tab displays a table listing all of the TFs that satisfy the search criteria or shows a form for submitting sequences. The user can select a TF from the list or upload custom DNA sequences. The ‘Results’ tab displays a table containing information on the analysed dataset, with a download link for the DNA sequence and shape data. The ‘Results’ tab also displays structural feature heat maps for individual sequences (Figure 1A), average heat maps for each shape parameter (Figure 1B), and the motif logo representing the PWM calculated using TFBS sequence information (Figure 1C). The TFBSshape interface dynamically updates the content under the tab pages, allowing the user to maintain a temporary customization throughout an analysis session.

### User interface for comparison of DNA shape profiles of two TF datasets

TFBSshape provides an interface for comparing two TFBS shape profiles from the database, or for comparing an uploaded TFBS dataset with a user-chosen reference TF dataset from the database. Under the ‘Selection’ tab, the user can initiate the selection to compare two TFs or to upload custom-aligned sequences. The ‘Refine’ tab then displays a form that guides the user through the selection of the two desired TF datasets, derived from either JASPAR or UniPROBE, or a form that enables the user to upload the sequences and select the desired reference TF dataset from the database. In this form, the user needs to specify the alignment of the two TF motifs by setting the reference positions for the compared datasets or the offset in nucleotide positions for the uploaded sequences. After this step, the user will find the comparison of DNA shape features under the ‘Results’ tab. The user can return to the ‘Refine’ tab to adjust the alignment.

**Figure 2.**
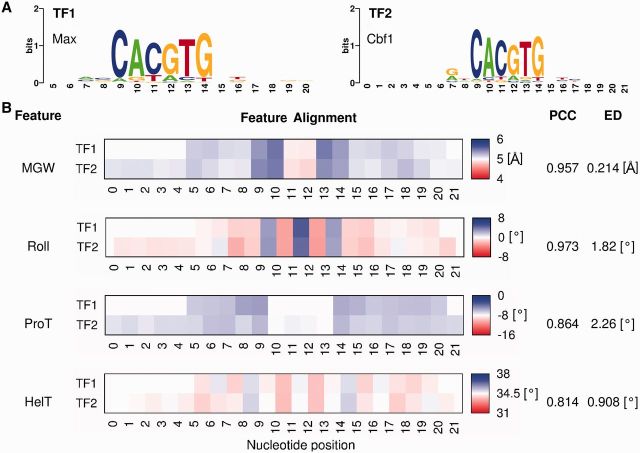
Example TFBSshape comparison of DNA shape preferences of two TF datasets from UniPROBE for the homologous TFs Max from mouse and Cbf1 from yeast. (**A**) PWMs calculated for Max and Cbf1 using all analysed TFBS sequences, with nucleotide positions numbered according to the user-determined alignment. (**B**) Using the chosen alignment, average heat maps for the four DNA shape features MGW, Roll, ProT and HelT are shown for Max (TF1) and Cbf1 (TF2). These shape profiles were quantitatively compared using PCC and ED.

As an example for this functionality of TFBSshape we compared UniPROBE datasets for the bHLH TFs Max and Cbf1, which are from mouse and yeast, respectively. The TFBSs were aligned based on the PWMs for both TFs (Figure 2A). Using the requested sequence alignment, TFBSshape visualized quantitative comparisons of average heat maps for the DNA shape features MGW, Roll, ProT and HelT, and provided Pearson’s correlation coefficients (PCC) and EDs as quantitative measures for the comparison (Figure 2B).

## BIOLOGICAL APPLICATIONS

### DNA shape preferences of human bHLH TFs

Paralogous TFs often bind to TFBSs with very similar and often identical core binding motifs, although they bind to different target sites in the genome to execute their specific *in vivo* functions. We previously showed that the yeast bHLH factors Cbf1 and Tye7 select distinct DNA shape features that contribute to their DNA binding specificities to genomic target sites, despite their strong preference for the CACGTG E-box as a shared core binding motif (17). TFBSshape can be used to compare structural features of TFBSs in analysing DNA binding specificities among TFs of the same family. Therefore, in this report, we extended our study to human bHLH factors. We analysed the TFBSs of Mad, Max and Myc derived from gcPBM experiments (27).

**Figure 3.**
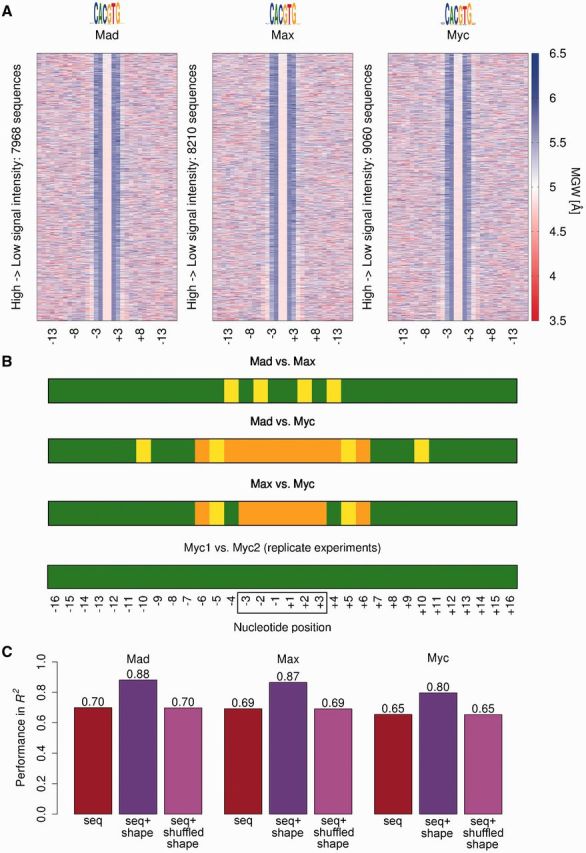
DNA shape preferences of human bHLH TFs. Heat maps illustrate MGW selections of (**A**) the Mad2-Max heterodimer (‘Mad’), the Max homodimer (‘Max’) and the c-Myc-Max heterodimer (‘Myc’). Sequence data were derived from gcPBM experiments (27) using 25% of the probes with highest signal intensities after removing probes with multiple TFBSs. (**B**) MGW preferences of the three TFs were compared, and nucleotide positions with significant MGW differences based on a K-S test were indicated for comparisons of Mad versus Max, Mad versus Myc and Max versus Myc (positions with different MGW distributions are shown in orange for *P* < 0.001 and yellow for *P* < 0.05; positions without significant differences are shown as green background). A replicate experiment for Myc verified that the gcPBM experiment and shape analysis did not detect any significant differences for Myc1 versus Myc2. The DNA shape features were symmetrized based on the palindromic E-box, which is located at the central positions –3 to +3 (frame). Detailed data for this analysis are provided in Supplementary Data (box plots in Supplementary Figure S2; *P*-values in Supplementary Table S1). (**C**) L2-regularized MLR and 10-fold cross-validation were used to test the accuracy of binding specificity predictions, showing that shape-augmented models (purple) outperformed specificity models using nucleotide sequence alone (brown) for all three human bHLH TFs. Adding randomly shuffled DNA shape features did not lead to the observed improvement (magenta).

Heat maps and box plots for MGW (Figure 3A, Supplementary Figure S2A–C), Roll, ProT and HelT (Supplementary Figures S3–S5) clearly indicated the unique structural features of the E-box. In addition, a Kolmogorov–Smirnov (K-S) significance test revealed that Mad and Max exhibit much more similar DNA shape preferences compared to the more distinct DNA binding specificity of Myc (Figure 3B, Supplementary Figures S2–S5). Whereas the E-box as a core binding motif is shared between all three TFs, differential DNA binding specificities can be detected through motif-based analysis of DNA shape preferences. These differences can be due to variations in the flanking sequences, as shown for Cbf1 and Tye7, or nucleotide variants within the E-box (Supplementary Table S1). To confirm the significance of the detected TFBS shape differences, we analysed a replicate experiment using Myc. The results indicated that the DNA shape features selected by the same TF in two independent gcPBM experiments were not distinct, according to K-S *P*-values (Figure 3B, Supplementary Figure S2D, Supplementary Table S1).

To further test whether the subtle differences in DNA shape features of the binding sites of the three paralogous bHLH TFs Mad, Max and Myc contribute to binding specificity beyond nucleotide sequence, we used L2-regularized MLR and 10-fold cross-validation to assess the prediction accuracy of models using sequence alone compared to a combination of sequence and shape parameters (MGW, Roll, ProT and HelT). We found that experimentally determined DNA binding specificities could be predicted with *R*^2^-values between 0.65 and 0.70, whereas a shape-augmented model reached *R*^2^-values between 0.80 and 0.88 (Figure 3C). Thus, by incorporating DNA shape features into binding specificity predictions, we achieved improvements of ∼26% for Mad, ∼26% for Max and ∼23% for Myc. These improvements indicate an important contribution of DNA shape features in protein–DNA recognition. In this model, DNA sequence and shape features were encoded using a strategy similar to our previous study of yeast bHLH TFs (16) but here we also considered variations within the E-box core motif. Using randomly shuffled shape parameters did not lead to any improvement over the sequence-based model (Figure 3C). These results clearly demonstrate the added value of shape-augmented descriptions of TFBSs in the modelling of DNA binding specificities.

### DNA shape preferences of Hox TFs in mouse

**Figure 4.**
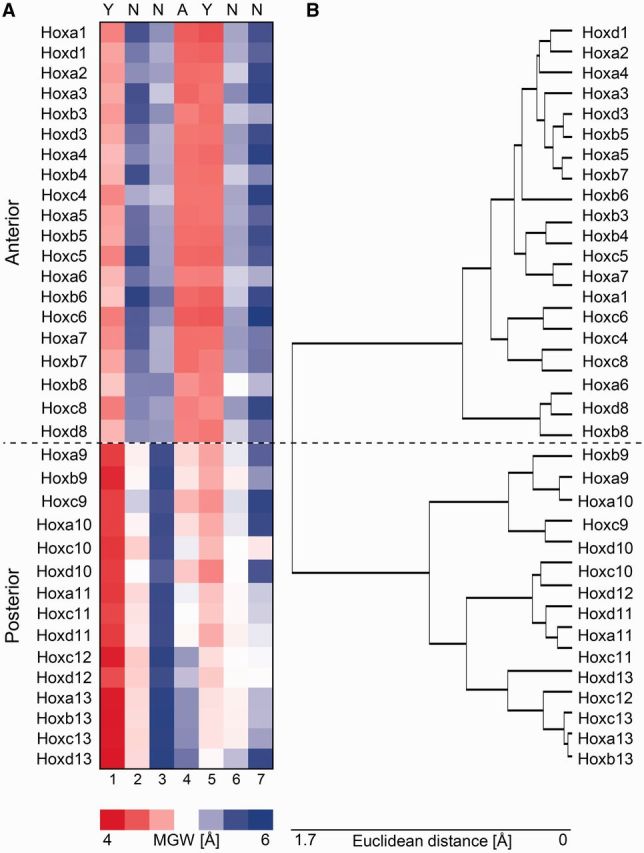
DNA shape features distinguish TFBSs of anterior and posterior Hox proteins in mouse. (**A**) Heat map illustrating average MGW profiles of binding sites preferred by mouse Hox TFs determined by universal PBM (28). (**B**) A dendrogram based on EDs between average MGW profiles of preferred TFBSs demonstrates the different DNA shape preferences of anterior and posterior Hox TFs in mouse.

We previously demonstrated that anterior and posterior *Drosophila* Hox proteins prefer distinct minor groove geometries (11,12), and recently analysed DNA shape preferences of mouse homeodomain TFs (13) derived from universal PBM experiments (28). Here, we show that the distinct DNA shape preferences of anterior and posterior Hox proteins hold for mouse, based on comparisons of their MGW (Figure 4A), Roll, ProT and HelT profiles (Supplementary Figure S6). Using EDs of MGW profiles, we generated a dendrogram revealing relationships between DNA binding specificities of mouse Hox proteins, and demonstrated clear distinctions in MGW preferences between anterior and posterior Hox TFs in mouse (Figure 4B), similar to the distinction previously observed for *Drosophila* (12). Thus, the TFBSshape database can be utilized to study relationships in DNA binding specificities of closely related TFs within protein families.

## CONCLUSIONS

We have demonstrated that augmenting existing motif databases with DNA shape features provides new insights into the mechanisms used by TFs to achieve DNA binding specificity. Analysing DNA shape preferences can help to differentiate between similar DNA binding specificities of paralogous TFs (17). Such studies can be generalized to compare DNA binding specificities of homologous TFs from different species. Comparisons of structural features of TFBSs could potentially reveal evolutionary relationships between TFs based on the shape of their DNA binding sites (13). Integrating TFBSshape with the motif databases JASPAR (25) and UniPROBE (26) makes DNA shape information readily available for known motifs. Whereas TFBSshape currently contains data for 23 species from the open-access motif databases JASPAR and UniPROBE, species-specific databases (33,34) can easily be integrated to expand the repertoire of datasets for comparative analysis of TF binding specificities. The availability of DNA shape features for TFBSs suggests many further applications, such as shape-augmented genome annotations (9) and TFBS predictions using DNA structural features (35–37).

## SUPPLEMENTARY DATA

Supplementary Data are available at NAR Online.

## FUNDING

USC-Technion Visiting Fellows Program, an Alfred P. Sloan Research Fellowship (to R.R.); National Institutes of Health (NIH) [U01GM103804 and R01HG003008] (in part to R.R.); PhRMA Foundation Research Starter Grant (to R.G.). Funding for open access charge: USC-Technion Visiting Fellows Program.

*Conflict of interest statement*. None declared.
